# Inhibition of CD147 Attenuates Stroke-Associated Pneumonia Through Modulating Lung Immune Response in Mice

**DOI:** 10.3389/fneur.2019.00853

**Published:** 2019-08-07

**Authors:** Rong Jin, Shan Liu, Min Wang, Wei Zhong, Guohong Li

**Affiliations:** Department of Neurosurgery, The Pennsylvania State University College of Medicine, Hershey, PA, United States

**Keywords:** CD147, stroke, pneumonia, IL-17A, IFN-γ

## Abstract

**Background and Purpose:** Acute ischemic stroke triggers a profound systemic and local immunodysfunction that increased the susceptibility to infections, especially stroke-associated pneumonia (SAP). Our previous study has shown that inhibition of CD147 ameliorates acute ischemic stroke, however, the role of CD147 in post-stroke lung infection has not been investigated.

**Methods:** C57BL/6 mice were subjected to transient (60 min) middle cerebral artery occlusion, and treated with anti-CD147 antibody (αCD147). Lung histological changes, vascular permeability, and pulmonary edema were determined. Bacterial burden in the lung tissue and Broncho alveolar lavage fluid (BALF) were measured. Lung leukocyte infiltration, circulating platelet-leukocyte aggregates, cell type-specific IL-17A, and IFN-γ expression in the lung were detected by flow cytometry.

**Results:** CD147 expression was markedly upregulated in the lung after stroke. αCD147 treatment significantly decreased the stroke-associated lung histological damages, bacterial load, vascular permeability and pulmonary edema. The protective effects by αCD147 treatment were associated with deceased lung inflammatory cell infiltration by reducing IL-17A expression in lung γδ T cells and attenuated bacterial load by enhancing IFN-γ expression in the lung NK1.1^+^ cells and CD4^+^ T cells. In addition, CD147 expression was also increased in the circulating platelets and leukocytes. Enhanced platelet-leukocyte aggregates following stroke was inhibited by αCD147 treatment.

**Conclusions:** Inhibition of CD147 ameliorates aberrant lung inflammatory and immune response and reduces bacterial infection after stroke. CD147 might represent a novel and promising therapeutic target for post-stroke lung infection.

## Introduction

Acute ischemic stroke not only produces local brain damage but also triggers a profound systemic immune dysfunction, which results in approximately 30% of patients suffering from stroke-associated pneumonia (SAP) within the first few days after stroke (1–3). Experimental studies have shown that prophylactic antibiotic treatment and/or inhibition of increased sympathetic activity using the β-adrenergic receptor blocker propranolol are effective for preventing and treating SAP (4–6). However, the clinical relevance of these treatments remain uncertain. Two recent large-scale clinical trials demonstrated that prophylactic antibiotic treatments did not reduce the incidence of mortality or SAP (7, 8). Moreover, beta-blocker therapy also did not reduce the risk for SAP, and had no direct effect on mortality at 7-days, even higher 30-days mortality (9). Thus, there is an urgent need to identify molecular signaling pathways that can be targeted with innovative therapies for SAP.

Cluster of differentiation 147 (CD147), a type I transmembrane glycoprotein of the immunoglobulin superfamily, is broadly expressed on different cell types, such as erythrocytes, leukocytes, platelets, endothelial cells and epithelial cells (10). Therapeutic targeting of CD147 has yielded encouraging results in experimental models of human diseases, such as rheumatoid arthritis, asthmatic lung inflammation, myocardial ischemia/reperfusion injury, autoimmune encephalomyelitis, and meningitis epidemics (11–15). CD147 was also involved in immune synapse formation, T cells activation and Th17 cells differentiation by modulation of cytokine production (16–18). Our recent study has demonstrated that inhibition of CD147 ameliorates acute ischemic stroke in mice (19). However, the role of CD147 in stroke-associated lung immune response and pneumonia has not been investigated. Thus, the present study was designed to investigate the role of CD147 on the lung immune response and infection following acute ischemic stroke. We examined the therapeutic potential of blocking CD147 and its mechanism of action in prevention of SAP in mice after focal cerebral ischemia/reperfusion injury.

## Materials and Methods

Details of materials and experimental procedures are available in Supplemental Material. All animal experiments were approved by the Institutional Animal Care and Use Committee at Penn State University College of Medicine.

### Stroke Model and Antibody Treatment

Mouse model of acute ischemic stroke was induced using transient (60 min) middle cerebral artery occlusion (MCAO) technique as we described previously (19, 20). Two hours after stroke, the mice were randomly assigned to the following treatment groups: a rat anti-mouse CD147 monoclonal antibody (1 mg/kg, Clone RL73.2, eBbioscience, named αCD147 mAb throughout this article) or isotype control antibody (rat IgG2a) administered via tail vein injection in 100 μL volume of phosphate buffered saline (PBS, pH 7.4). For randomization, the web tool www.randomizer.org was used. The dose of 1 mg/kg per day for αCD147 was selected based on our previous studies (19). In the 24-h survival experiments, a single dose of antibody was given at 4 h after onset of ischemia. In the 72-h survival experiments, antibody treatment was initiated at 4 or 8 h, and repeated at 24 and 48 h after onset of ischemia. The number of animals used in each experimental group was summarized in Supplementary Table 1.

### Infarcts Volumes and Neurological Deficits

Infarct volumes were measured in cresyl violet stained coronal sections on day 3 after MCAO, as previously described (20). The modified Bederson score (global neurological function) was performed by a blinded investigator (21).

### Lung Vascular Permeability and Water Content Assays

Lung vascular permeability was evaluated by Evans Blue dye (EB) method as described previously (22). The water content was calculated as = (wet weight-dry weight)/wet weight × 100%.

### Bacteriological Analysis

Mouse Bronchoalveolar lavage fluid (BALF) and lung tissues were collected. The number of cells and protein concentrations in the BALF were measured. Then, bacterial burden within the BALF and lung tissue homogenates were determined using brain-heart infusion (BHI) agar cultures as described previously (20, 23).

### Lung Histological and Immunohistochemical Assessment

Lung sections were stained with H&E and graded on a scale from (1) normal, (2) focal (<50% lung section) interstitial congestion and inflammatory cell infiltration, (3) diffuse (>50% lung section) interstitial congestion and inflammatory cell infiltration, (4) focal (<50% lung section) consolidation and inflammatory cell infiltration, to (5) diffuse (>50% lung section) consolidation and inflammatory cell infiltration, as previously described (24). Immunohistochemistry was performed as described previously (19).

### Western Blot and Enzyme-Linked Immunosorbent Assays (ELISA)

Mouse plasma and lung tissues were collected for analysis. The concentrations of IL-17A and IFN-γ were measured using specific ELISA kits according to manufacturers' protocol. Western blotting were performed as described previously (19).

### Flow Cytometry

Mouse blood and lung tissues were collected. The lung infiltrating cells were isolated with enzymatic digestion method as previously (25). Blood samples and isolated infiltrating cells were stained with antibody cocktails and run on a BD Accuri C6 Flow Cytometry. Data were analyzed by FlowJo software. The antibodies are listed as Supplementary Table 2.

### Quantitative Real-Time PCR

Mouse lung tissues were collected. Total RNA was extracted by using the TRIzol extraction kit (Life Technologies). Expression levels of chemokines (CXCL1 and CXCL2) were determined by quantitative real-time RT-PCR with a Bio-Rad thermocycler and an SYBR green kit (Bio-Rad) according to the recommendations of the manufacturer. Mouse Gapdh was used as reference gene. The primers had the following sequences: CXCL1 forward 5′-TCGATGGTAGTTCAGTTCTGCT-3′ and reverse 5′-TCGCACAACACCCTTCTACT-3′, CXCL2 forward 5′-CCCTGCCAAGGGTTGACTTC-3′ and reverse-5′- GGGGCTTCAGGGTCAAGG-3′, Gapdh forward 5′-GCGAGATCCCGCTAACATCA-3′, and reverse 5′-CTCGTGGTTCACACCCATCA-3′. All samples were run in triplicate. Relative levels of mRNA were normalized to the mouse housekeeping gene, and the results are expressed as fold change vs. the sham controls.

### Statistical Analysis

All results were expressed as mean ± SD except for ordinal lung histopathologic scores and Bederson score that were depicted as scatter plots. Graphpad Prism 5 software package was used for statistical analysis. Unless otherwise indicated, multiple comparisons were made using a 1-way ANOVA followed by the Bonferroni *post-hoc* test. If only 2 groups were compared, unpaired, 2-tailed Student *t*-test was applied. Non-parametric lung histopathologic scores and Bederson scores were compared by Kruskal-Wallis test with *post-hoc* Dunn corrections. *P* < 0.05 was considered statistically significant. All data were analyzed in a blinder manner.

## Results

### Inhibition of CD147 Attenuates Post-stroke Lung Damage

**Figure 1:**
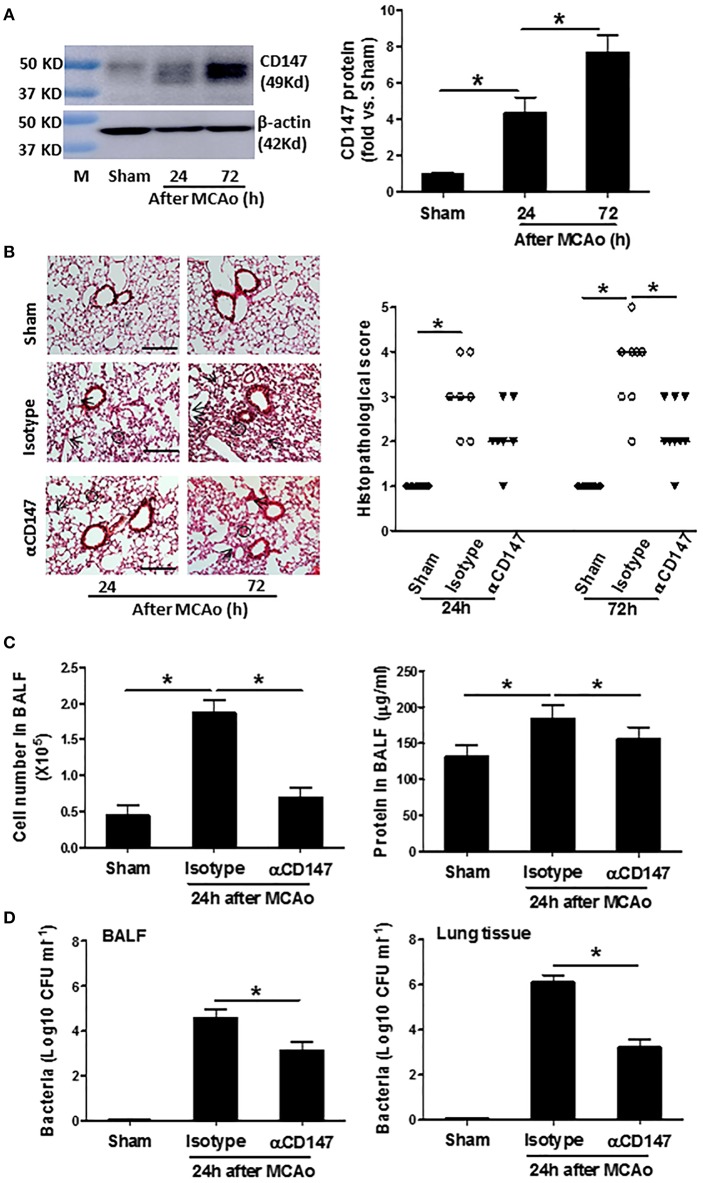
Inhibition of CD147 attenuates post-stroke pneumonia. **(A)** Representative images of western blots showing CD147 protein levels in the lung tissues and semi-quantitative analysis of immunoblots. M indicates protein marker. *n* = 4 per group. **(B)** Left panel: representative images of hematoxylin and eosin-stained lung sections. Black circles indicate the patchy areas of cellular consolidation. Arrows indicate the intra-alveolar infiltrates. Bar = 200 μm. Right panel: quantitative analysis of histopathological lung injury scores. Bars are median values. *n* = 6–9 per group. **(C)** The number of cells (left panel) and levels of protein concentration (right panel) in BALF at 24 h after stroke. *n* = 6 per group. **(D)** Quantitative analysis of bacterial loads by measuring colony-forming units (CFU) in BLAF (left panel) and lung tissue homogenate (right panel) cultures in indicated groups at 24 h after stroke. *n* = 6 per group. ^*^P < 0.05.

Western blot analysis showed that CD147 protein expression in the lung tissue was elevated significantly at 24 h, and continued to increase up to 72 h after stroke (The full images of western blot were available in Supplementary Figure 1) (Figure 1A). The unglycosylated CD147 has a molecular weight 27–29 kDa, whereas the glycosylated form has a molecular weight between 43 and 66 kDa (26). This variance could be attributed to differential post-translational modification in different cell types. We detected multiple bands of CD147 protein in the lung tissue, suggesting that they might derive from different cell types in the lung after stroke. In addition, flow cytometry data showed that the CD147 expression was significantly increased in the isolated lung neutrophils and Ly6C^Hi^ monocytes/macrophages after stroke (Supplementary Figure 2).

αCD147 treatment significantly decreased the development of post-stroke lung damage (Figure 1B). Histological examination revealed typical signs of bacterial pneumonia: lung consolidation, thickened alveolar septa and intra-alveolar inflammatory infiltrates in the isotype-treated mice at 24 h after stroke, and continued to development to 72 h (Figure 1B), as previously described (20). But no signs of pneumonia was found in the lung sections of sham mice (Figure 1B), indicating enhanced susceptibility to lung infection resulted from stroke but not from surgical stress. Moreover, the number of cells and levels of protein concentration in the BALF were significantly increased after stroke, but these increases were inhibited in αCD147-treated mice compared with isotype control group (Figure 1C). Furthermore, microbiological analysis revealed significant bacterial loads in the BALF and lung tissue homogenate cultures of all mice (*n* = 12) at 24 h after stroke (Figure 1D). In contrast, sample cultures from sham-operated mice remained sterile (Figure 1D). Importantly, αCD147 treatment profoundly reduced bacterial growth in the BALF (Figure 1D) and lung tissue cultures (Figure 1D) after stroke. In addition, no bacterial growth was observed in the blood cultures of either sham or MCAO mice (data not show), consistent with previous reports in C57Bl6 mouse strain (20, 27).

### Inhibition of CD147 Attenuates Post-stroke Lung Vascular Permeability and Edema Through Inhibition of MMP-9 Activity

**Figure 2:**
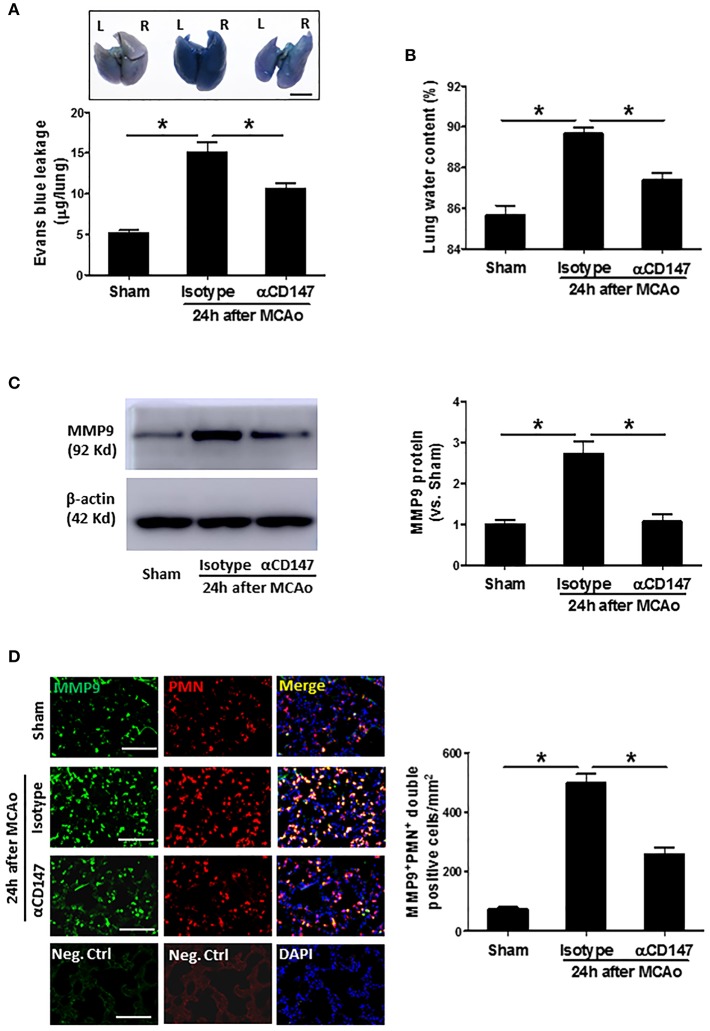
Inhibition of CD147 attenuates post-stroke lung vascular permeability and edema through inhibition of MMP-9 activity. **(A)** Representative pictures and quantitative analysis of Evans blue leakage in the lung tissue at 24 h after stroke. L: left lung (one lobe), R: right lung (four lobe). Bar = 5 mm. *n* = 6 per group. **(B)** Lung water content at 24 h after stroke. *n* = 6 per group. **(C)** Representative images of western blots showing MMP-9 protein levels in the lung tissues and semi-quantitative analysis of immunoblots. *n* = 4 per group. **(D)** Double immunostaining for MMP-9 and neutrophils in the lung sections in indicated groups at 24 h after stroke (left panel). Neg.Ctrl indicates negative control. Bar = 100 μm. The number of the cells double positively stained for neutrophils and MMP-9 per mm^2^ was counted (right panel). *n* = 6–7 per group. ^*^P < 0.05.

The lung vascular permeability was assessed by measuring extravasation of EB. The amount of extravasated EB dye (Figure 2A) within the lung tissue was significantly increased at 24 h after stroke compared with sham controls. However, it was reduced in the αCD147-treated mice compared with isotype-treated mice (Figure 2A). Correspondingly, pulmonary edema was significantly reduced in αCD147-treated mice compared with isotype-treated mice (Figure 2B). MMPs, in particular MMP-9, is involved in the pathogenesis of various pulmonary inflammatory diseases and associated with pulmonary edema (28). Western blot showed MMP-9 expression was significantly increased in the lung after stroke (The full images of western blot were available in Supplementary Figure 1) (Figure 2C), which was mainly derived from infiltrated neutrophils (Figure 2D) as determined by double immunostaining. Importantly, it was significantly abolished by αCD147 treatment (Figures 2C,D).

### Inhibition of CD147 Modulates Changes in Leukocyte Subpopulations in the Lung After Stroke

**Figure 3:**
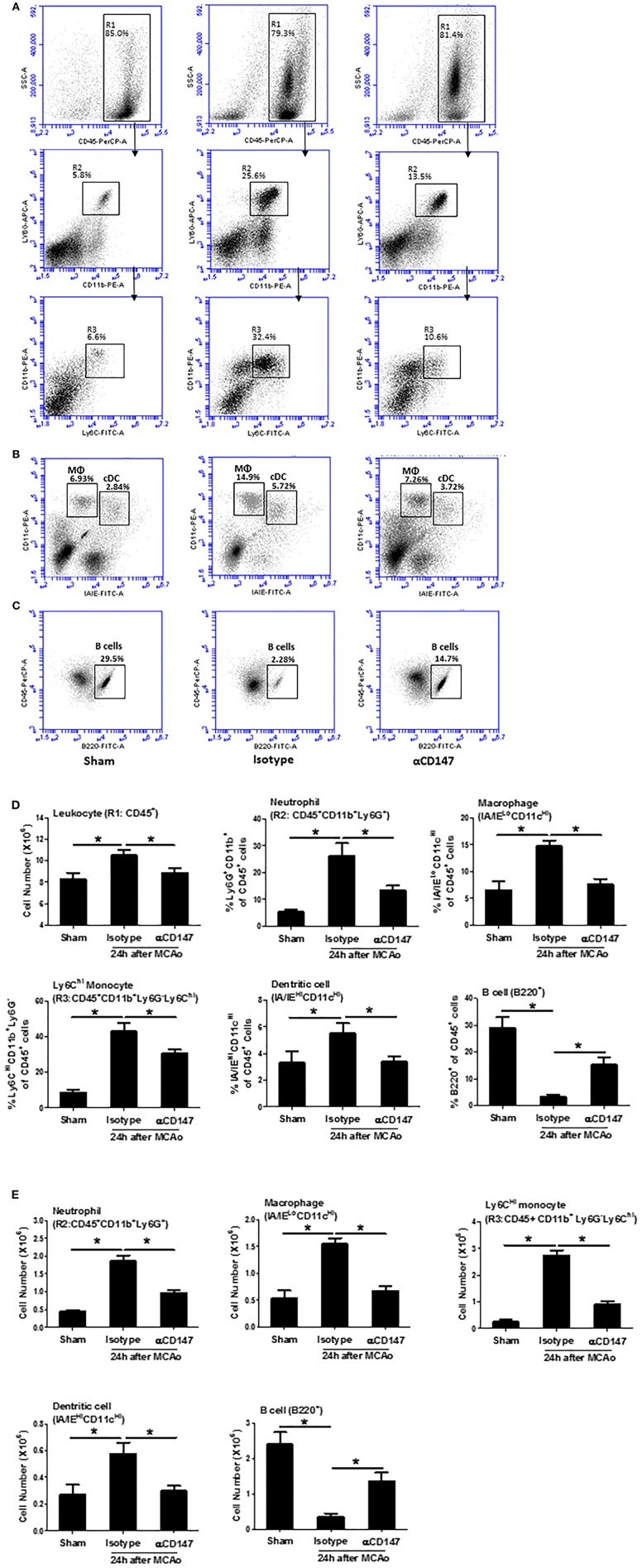
Inhibition of CD147 modulates the changes of leukocyte subpopulations in the lung after stroke. **(A)** Representative flow cytometric dot plots of R1: total leukocytes (CD45^+^), R2: neutrophils (CD45^+^CD11b^+^Ly6G^+^), and R3: ly6C^Hi^ monocytes (CD45^+^CD11b^+^Ly6G^−^Ly6C^Hi^) in the isolated lung cells from indicated groups. Lung infiltrating cell was isolated using enzyme digestion method at 24 h after stroke. **(B)** Representative flow cytometric dot plots of lung conventional dendritic cell (cDC, identified as CD11c^Hi^/IA/IE^Hi^) and macrophage (MΦ, CD11c^Hi^/IA/IE^Lo^) populations. **(C)** Representative flow cytometric dot plots of B cell population (B220^+^). **(D,E)** The number and frequency of cell subpopulations in the indicated groups. *n* = 6 per group. ^*^P < 0.05.

Leukocyte subpopulations in the lung tissues were analyzed by flow cytometry at 24 h after stroke. Figures 3A–C showed a gating strategy for identifying immune cell subpopulations in the lung, and representative flow cytometric dot plots in the indicated groups. The number of total leukocytes (CD45^+^), and the frequencies (Figure 3D) and absolute cell numbers (Figure 3E) of neutrophils (CD45^+^CD11b^+^Ly6G^+^), Ly6C^Hi^ monocytes (CD45^+^CD11b^+^Ly6G^−^Ly6C^Hi^), dendritic cells (CD45^+^CD11c^Hi^IA/IE^Hi^), and macrophages (CD45^+^CD11c^Hi^ IA/IE^Lo^) were significantly increased in the lungs of the isotype-treated group compared with sham controls, but these changes were attenuated in the αCD147-treated group compared with the isotype-treated group (Figure 3D). Previous study showed that lung B cells play an important role in protecting against pneumonia via producing IgM (29). Interestingly, compared with sham mice, the frequency and absolute number of B cells in the lung were significantly decreased after stroke, but these decreases were significantly attenuated in αCD147 treatment group (Figures 3D,E).

### Inhibition of CD147 Decreases Circulating Platelet-Leukocyte Aggregates After Stroke

**Figure 4:**
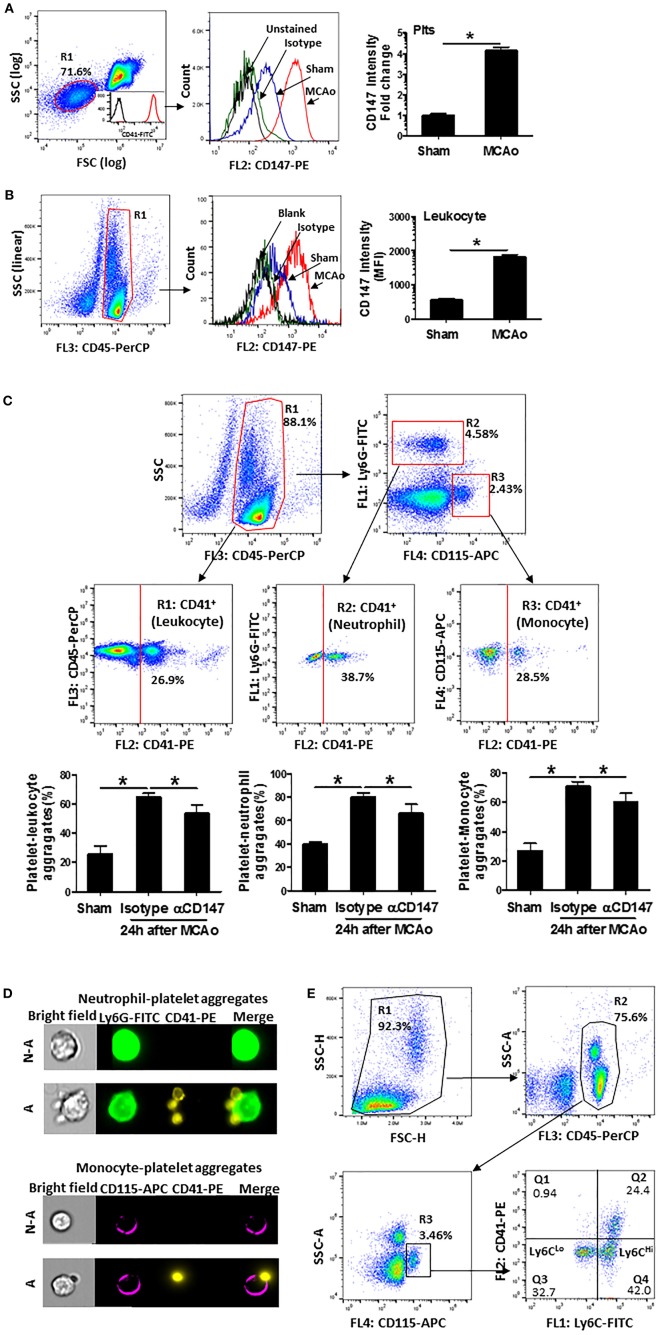
Inhibition of CD147 decreases circulating platelet-leukocyte aggregates after stroke. **(A)** Representative flow cytometry strategy for gating platelet population (R1, left), which was confirmed by >98% CD41 positive staining (insert histogram). Histogram of flow cytometry showing CD147 expression on the platelets in the indicated groups (mid) and quantitative analysis of platelets CD147 expression (right). Data are expressed as mean fluorescence intensity (MFI). *n* = 4 per group. **(B)** Representative flow cytometry strategy for gating leukocyte population (R1: CD45^+^, left), histogram of flow cytometry showing CD147 expression on the leukocytes in the indicated groups (mid) and quantitative analysis of leukocytes CD147 expression (right). *n* = 4 per group. **(C)** Representative flow cytometry strategy for gating leukocyte subpopulations from the blood cells, R1: CD45^+^ total leukocyte, R2: neutrophil (CD45^+^ly6G^+^), R3: monocyte (CD45^+^CD115^+^) and quantitative analysis of circulating platelet-leukocyte aggregates (% total CD45^+^ cells), platelet-neutrophil aggregates (% total CD45^+^Ly6G^+^ cells) and platelet-monocyte aggregates (% total CD45^+^CD115^+^ cells) (right) in the indicated groups. *n* = 6 per group. **(D)** Representative brightfield (BF) and fluorescent images, acquired by imageStream; Ly6G^+^ neutrophils (green) aggregated to platelets (CD41^+^ yellow) (upper panel); monocyte (CD115^+^ purple) aggregated to platelets (CD41^+^ yellow) (down panel). N-A, cell non-aggregated with platelet; A, cell aggregated with platelet. Cells are from blood sample. **(E)** Representative flow cytometry strategy for identifying platelet- Ly6C^Lo^ and/or Ly6C^Hi^ monocyte aggregates from the blood cells. Results showed that predominant monocytes in platelet-monocyte (CD41^+^CD115^+^) aggregates were Ly6C^Hi^ cells.

Enhanced platelet-leukocyte aggregates are a common mechanism for promoting leukocyte recruitment to the site of inflammation (30). Thus, we used flow cytometry to analyze CD147 expression and circulating platelet-leukocyte aggregates in whole blood at 24 h after stroke. The expression of CD147 was markedly increased both in platelets (Figure 4A) and leukocytes (Figure 4B) at 24 h after stroke. Figure 4C showed the flow cytometry strategy for gating and identifying platelet aggregated leukocytes. Compared with sham mice, the circulating levels of platelet-leukocyte (CD41^+^CD45^+^), platelet-neutrophil (CD41^+^Ly6G^+^) and platelet-monocyte (CD41^+^CD115^+^) aggregates were significantly increased after stroke (Figure 4C). However, the increases in αCD147 treatment group were significantly lower than that in isotype treatment group (Figure 4C). ImageStream analysis was used to confirm the platelet-leukocyte (neutrophil and monocyte) aggregates (Figure 4D). In addition, the monocytes in the circulating platelet-monocyte (CD41^+^CD115^+^) aggregates were almost exclusively Ly6C^Hi^ cells (Figure 4E).

### Inhibition of CD147 Reduces Lung and Plasma IL-17A Levels After Stroke

**Figure 5:**
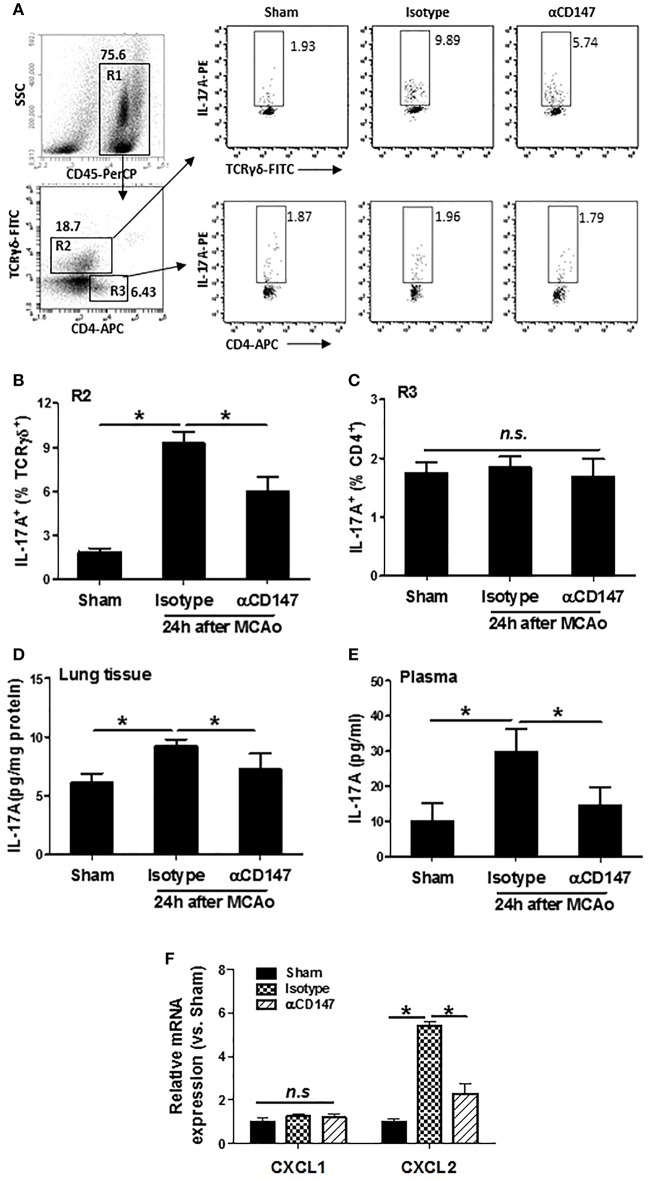
Inhibition of CD147 reduces lung and plasma IL-17A expression after stroke. **(A)** Representative flow cytometry strategy for gating and identifying IL-17A-producing γδT cells (R2:CD45^+^TCR-γδ^+^CD4^−^) and CD4^+^ T cells (R3:CD45^+^TCR-γδ^−^CD4^+^). Lung-infiltrating cell was isolated at 24 h after stroke. **(B,C)** Quantification of lung IL-17A-producing γδT cells **(B)** and CD4^+^ T cells **(C)** in the indicated groups. *n* = 6 per group. **(D,E)** ELISA assay of IL-17A protein concentrations in the lung tissue **(D)** and plasma **(E)** at 24 h after stroke. *n* = 6 per group. n.s. indicates not statistically significant. ^*^P < 0.05. **(F)** Real-time RT-PCR analysis of mRNA expression of the chemokines (CXCL1 and CXCL2) in the lung after stroke. *n* = 5 per group. n.s. indicates not statistically significant. ^*^P < 0.05.

Interleukin 17A (IL-17A), a major inducer of the chemokines, is involved in leukocyte recruitment (31). The expression of IL-17A in the lung γδ T (γδT-17) cells and CD4^+^ T (Th17) cells was determined by flow cytometry. Data showed that αCD147 treatment significantly decreased the stroke-induced upregulation of IL-17A in the lung γδT-17 cells (Figures 5A,B). However, its expression in CD4^+^ T cells was no significant changes between the three groups (Figures 5A,C). We also measured the concentration of IL-17A in the lung and plasma with ELISA kit. The results showed that IL-17A protein concentration was markedly increased in the lung tissue and plasma in isotype-treated mice compared with sham controls (Figures 5D,E), but they were largely attenuated in αCD147-treated mice (Figures 5D,E). IL-17A plays a critical role in the activation and recruitment of neutrophils to the site of inflammation mainly through the induction of CXC chemokines (CXCL1 and CXCL2) (32, 33). Next, we examined the mRNA expression of CXCL1 and CXCL2 in the lung after stroke. The data showed that the expression of CXCL2 was significantly increased after stroke. However, it was dramatically inhibited by αCD147 treatment (Figure 5F). The expression of CXCL1 was no significant differences between the three groups (Figure 5F).

### Inhibition of CD147 Increases Lung, but Not Plasma IFN-γ Levels After Stroke

**Figure 6:**
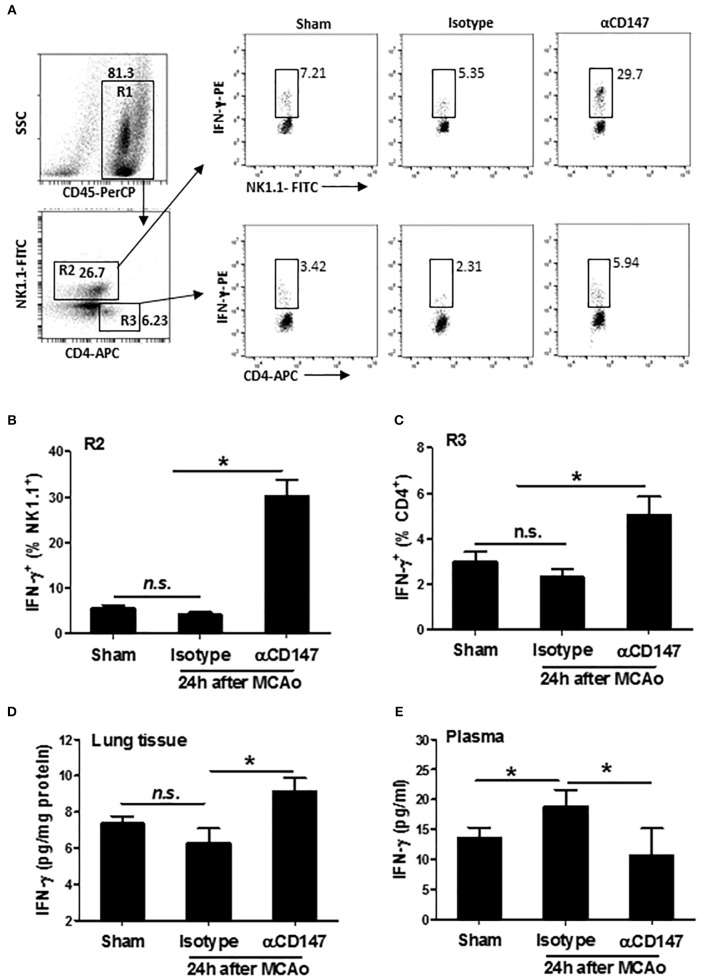
Inhibition of CD147 increases lung, but not plasma IFN-γ expression after stroke. **(A)** Representative flow cytometry strategy for gating and identifying IFN-γ-producing NK1.1 cells (R2: CD45^+^NK1.1^+^CD4^−^) and CD4^+^ T cells (R3: CD45^+^NK1.1^−^CD4^+^). Lung infiltrating-cell was isolated at 24 h after stroke. **(B,C)** Quantification of lung IFN-γ-producing NK1.1^+^ cells **(B)** and CD4^+^ T cells **(C)** in the indicated groups. *n* = 6 per group. **(D,E)** ELISA assay of IFN-γ protein concentration in the lung tissue **(D)** and plasma **(E)** at 24 h after stroke. *n* = 6 per group. n.s. indicates not statistically significant. ^*^P < 0.05.

Interferon gamma (IFN-γ), an important cytokine in the host defense against infection, which is derived mainly from NK and CD4^+^ T cells during pneumonia (34). Next, we examined the expression of IFN-γ in the lung NK and CD4^+^ T cells after stroke. The data showed that the expression of IFN-γ was increased both in NK1.1^+^ cells and CD4^+^ T cells in αCD147-treated mice compared with isotype-control mice (Figures 6A–C). There was no significant changes between isotype-treated mice and sham controls (Figures 6A–C). Consistent with these findings, the results of ELISA showed that the IFN-γ protein concentration in the lung tissue was no significant changes in isotype-treated mice at 24 h after stroke compared with sham controls. However, it was significantly increased in αCD147-treated mice (Figure 6D). The content of IFN-γ protein in the plasma was markedly increased in isotype-treated mice compared with sham controls (Figure 6D). However, αCD147 treatment significantly decreased the content of IFN-γ protein in the plasma after stroke (Figure 6E).

### Delayed Inhibition of CD147 Does Not Affect Stroke Injury, but Attenuates Post-stroke Lung Damage

**Figure 7:**
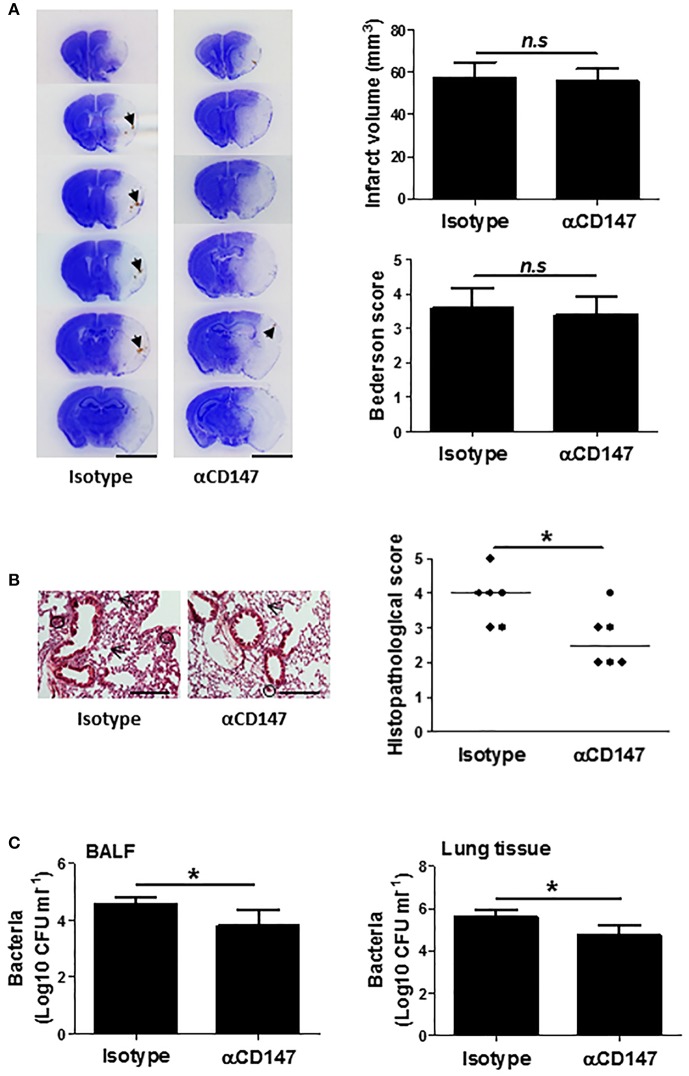
Delayed inhibition of CD147 does not affect brain injury, but attenuates post-stroke lung damage. **(A)** Representative images of cresyl violet-stained brain coronal sections of mice (left panel). Blue color: intact tissues, white color: infarct tissues. Arrows indicate spontaneous hemorrhagic transformation. Bar = 5 mm. Infarct volume and Neurological deficits (Right panel). *n* = 6 per group, n.s. indicates not statistically significant. **(B)** Representative images of hematoxylin and eosin-stained lung sections (Left panel). Black circles indicate the patchy areas of cellular consolidation. Arrows indicate the intra-alveolar infiltrates. Bar = 200 μm. Quantitative analysis of histopathological lung injury scores (Right panel). Bars are median values. *n* = 6 per group. **(C)** Quantitative analysis of bacterial loads by measuring colony-forming units (CFU) in BLAF (left panel) and lung tissue homogenate (right panel) cultures in indicated groups at 3 days after stroke. *n* = 6 per group. ^*^P < 0.05.

Infarct size is known to be associated with the incidence and severity of post stroke pneumonia (35, 36). Thus, we further determined whether the effects of anti-CD147 treatment on lung is independent of brain pathology, which couldn't be concluded from the above experiments with the early αCD147 treatment (initiated at 4 h after stroke onset) due to the previously shown neuroprotective effects (19). To address this issue, the mice were subjected to delayed αCD147 treatment initiated at 8 h after stroke onset. This delayed treatment regimen was used because very few treatments initiated beyond 6 h of stroke onset could substantially reduce infarct size in acute ischemic stroke experimental models. As expected, there were no significant differences in infarct volumes and neurological deficits between isotype- and delayed αCD147-treated groups at 3 days after stroke (Figure 7A). However, we observed a relatively higher incidence of spontaneous hemorrhagic transformation in the isotype-treated group (4 of 6 mice) than that in αCD147-treated group (2 of 6 mice). Our data showed that the delayed αCD147 treatment significantly decreased post-stroke lung damage (Figure 7B) and bacterial loads in the BALF and lung tissues (Figure 7C).

## Discussion

The present study for the first time demonstrates that CD147 plays a critical role in post-stroke pneumonia. Antibody blockade of CD147 substantially attenuates lung susceptibility to bacterial infection and lung leukocyte infiltration and thereby ameliorates stroke-associated pneumonia (SAP) and lung histological damage after stroke.

The lungs are particularly vulnerable in patients with severe brain damage, including ischemic and hemorrhagic strokes (37, 38). It has been shown that ischemic stroke caused excessive leukocyte infiltration into lung and reduced alveolar macrophage phagocytic capability, which contributes importantly to poststroke lung injury (39). Innate immune cells, primarily neutrophils and monocytes, are rapidly recruited to the infected sites to control and clear invading pathogens (40). However, excessive lung leukocyte infiltration can severely compromise pulmonary function by release of various toxic factors including proteases, cationic polypeptides, proinflammatory cytokines, and reactive oxygen species (ROS). Indeed, inhibition of aberrant lung inflammatory response has been shown to protect against lung injury (41). Previous studies have demonstrated that inhibition of CD147 markedly reduces leukocyte infiltration into inflamed tissues in several mouse models, such as asthmatic lung inflammation, multiple sclerosis, autoimmune encephalomyelitis myocardial infarction and ischemic stroke (12–14, 19). In the present study, we demonstrated that inhibition of CD147 profoundly reduced lung local inflammatory responses following stroke, as evidenced by reduced leukocyte infiltration into lung tissue and reduced cell number and protein levels detected in the BALF. Increased circulating platelet-leukocyte aggregates represent a common mechanism for promoting leukocyte recruitment in many inflammatory diseases, including myocardial infarction, acute lung injury, and ischemic stroke (30, 42–44). In the present study, we found that CD147 expression was upregulated in circulating platelets and leukocytes after stroke, and αCD147 treatment significantly decreased the levels of circulating platelet-leukocyte aggregates, which likely contribute to reduced lung leukocyte infiltration after stroke.

IL-17A is a major pro-inflammatory cytokine that has been shown to act as a key mediator of leukocyte recruitment (31). IL-17A can activate several signaling cascades, leading to the induction of CXC chemokines, such as Cxcl1 and Cxcl2, which in turn generate chemotactic signals for leukocyte recruitment to the site of inflammation (31, 41, 45). Overexpression of IL-17A or the administration of recombinant IL-17A in the lung promotes the production and release of CXC chemokines and neutrophil recruitment (46). In contrast, selective inhibition of IL-17A signaling reduces ischemia/reperfusion-induced brain damage in mice (47–49). γδ T cells and CD4^+^ Th17 T cells, both are enriched in the lung tissue, are considered to be the major cellular source of IL-17A (31, 50). In the present study, we found that the expression of IL-17A was increased at 24 h in the lung tissue after stroke, which was mainly derived from γδ T cells, but not from CD4^+^ Th17 cells. More importantly, we found that the increased IL-17A-expressing γδ T cells, as well as the increased IL-17A protein levels in both lung tissue and plasma after stroke, were significantly reduced in the αCD147-treated group compared with the isotype-treated group. In mice, γδ T cells can be characterized by the expression of CCR6 and either Vγ1, Vγ4, or Vγ6. It has been shown that Vγ6^+^/CCR6^+^γδ T cells are the major producer of IL-17A in the ischemic brain (51). Which subsets of γδ T cells are the main producer of IL-17A in the lung after stroke and its potential modulation by CD147 warrant further investigation.

IFN-γ is a pivotal mediator in host defense against a variety of infectious agents. It is produced mainly by NK cells and antigen-activated T cells. It has been shown that an impaired early NK- and T-cell response, particularly reduced IFN-γ production, is critical for stroke-induced defect of the antibacterial defense (5). Adoptive transfer of IFN-γ-producing NK and T cells or early treatment with recombinant IFN-γ inhibited bacteremia and pneumonia (5, 31). However, IFN-γ may act as a double-edged sword in the pathophysiology of stroke. Although IFN-γ helps to protect against bacterial infection and to prevent the stroke-associated pneumonia, elevated levels of IFN-γ in the circulation also could exacerbate ischemic brain injury through enhancing systemic inflammation (52). It has been reported that antibody blockade of CD147 stimulated IFN-γ production in the CD3/CD28 antibodies-activated human T cells (16). In the present study, we found that αCD147 treatment had dual beneficial effects, i.e., it increased the IFN-γ levels in the lung tissue that are mainly derived from NK1.1^+^ cells and CD4^+^ T cells, whereas reduced the IFN-γ levels in the circulating plasma. The mechanisms by which αCD147 treatment differentially alters the IFN-γ in the lung and blood remain unknown and warrant further investigation.

Stroke size is associated with the incidence and severity of SAP (35, 36). Our previous study has shown that inhibition of CD147 significantly reduced stroke size (19). This raises an important question whether the protection against SAP by αCD147 treatment is simply a result of smaller stroke size. To address this issue, the mice were subjected to αCD147 treatment initiated at 8 h after stroke onset. We found that the delayed αCD147 treatment did not alter stroke size compared with the isotype-treated control, but was still effective in reducing bacterial loads and lung tissue damage at 3 days after stroke. These findings suggest that αCD147 treatment attenuates SAP via mechanisms that are likely independent of the stroke size.

## Conclusions

The present study demonstrates that inhibition of CD147 ameliorates SAP through inhibiting aberrant lung immune response and bacterial infection after stroke. Thus, CD147 might represent a novel and promising target for preventing/treating SAP.

## Data Availability

All datasets generated for this study are included in the manuscript and/or the Supplementary Files.

## Ethics Statement

All animal experiments were approved by the Institutional Animal Care and Use Committee at Penn State University College of Medicine.

## Author Contributions

RJ, SL, MW, and WZ performed the experiments and the data analysis. RJ and GL designed and supervised the study. RJ wrote the manuscript. GL revised the manuscript.

### Conflict of Interest Statement

The authors declare that the research was conducted in the absence of any commercial or financial relationships that could be construed as a potential conflict of interest.
