# Supplementary material for: Inhibition of CD147 Attenuates Stroke-Associated Pneumonia Through Modulating Lung Immune Response in Mice
Source: Front Neurol. 2019 Aug 7;10:853. doi: 10.3389/fneur.2019.00853 (PMC6692478; doi:10.3389/fneur.2019.00853)
Supplement: Supplementary file 1 [file Data_Sheet_1.docx]

**SUPPLEMENTAL MATERIAL.**

**I. Supplemental Methods:**

**Mouse Stroke Model and Antibody Treatment**

All animal experiments were approved by the Institutional Animal Care and Use Committee at Penn State University College of Medicine. Male C57BL/6 mice (8-10 weeks, Jackson Laboratories, Bar Harbor, ME) were subjected to transient (60 minutes) middle cerebral artery occlusion (tMCAO) using the intraluminal filament occlusion method as we described previously (Jin et al., 2010; Jin et al., 2011 and Jin et al., 2017). Briefly, a 6-0 silicone-coated nylon monofilament (Doccol Corp.) was introduced from the left external carotid artery into the internal carotid artery and advancing it to the origin of the MCA until cerebral blood flow was abruptly reduced. After 60 minutes of the occlusion, the restoration of blood flow to the MCA territory was made by withdrawal of the nylon suture. Only animals that exhibited a reduction in cerebral blood flow >85% during MCAO and a cerebral blood flow recovery by >80% after 15 minutes of reperfusion were included in the study. Sham-operated animals underwent anesthesia and exposure of the arteries without tMCAO induction. Rectal temperature was maintained at 37.0 ± 0.5°C throughout the surgical procedure with a feedback-regulated heating pad. Sustained-release formulation of buprenorphine (SB, 2.2 mg/kg) and 1ml pre-warmed 0.9% saline were injected subcutaneously before surgery and repeated every 24 hours for pain management and fluid loss compensation. Regional cerebral blood flow (CBF) was monitored in all stroke animals before ischemia, 5 minutes after tMCAO and 15 min after reperfusion using laser Doppler flowmetry (MSP300XP; AD Instruments Inc). The exclusion criteria were as follows: (1) Death within 24 h after tMCAO; (2) Subarachnoid hemorrhage (as macroscopically assessed - brain sampling); (3) Bederson score  =  0 (24 hours after tMCAO). Two hours after tMCAO, animals were randomly assigned to the following groups: a rat anti-mouse CD147 monoclonal antibody (RL73.2, eBbioscience, named αCD147 mAb throughout this article) or isotype control antibody (rat IgG2α) administered via tail vein injection in 100 ul volume of PBS. Antibody treatment was initiated at 4 or 8 hours and repeated at 24 hours and 48 hours after onset of ischemia. This anti-CD147 antibody has been well characterized to block CD147 function in various mouse models (Jin et al 2017., Gwinn et al., 2006; Seizer et al., 2011; Agrawal et al., 2011; Agrawal et al., 2012; Damsker et al., 2009). The number of animals used in each experimental treatment group was summarized in Suppl Table I.

**Infarct volumes and neurological deficits**

On day 3 after MCAO, the mice were euthanized by deep anesthesia and then perfused transcardially with 30 mL of PBS followed by cold 4% paraformaldehyde in PBS. The brains were collected and incubated overnight in the 4% paraformaldehyde solution and then transferred into 10%, 20%, and 30% sucrose solution until they sank. Then, coronal sections (40-μm thick) were serially cut using a cryostat, starting from the frontal pole through the entire brain, and 6 coronal sections at 1 mm intervals (relative to bregma: +2, +1, 0, −1, −2, −3 mm) were selected and stained with 0.1% cresyl violet solution (pH 3.8) at 37 °C for 5 minutes. The areas of the uncorrected infarcted area and the total areas of both hemispheres were measured for each slice by a computerized National Institutes of Health image analysis system. Corrected infarct area in a slice was calculated by subtracting the area of normal tissue in the ipsilateral hemisphere from the total area of the contralateral hemisphere. Total corrected infarct volume was then calculated by multiplying the area by the slice thickness and summing the volumes from all slices. All measurements were performed by 2 blinded researchers, and the mean of their results was calculated.

Before euthanasia, the modified Bederson score (Bederson et al., 1986) was detected to determine global neurological function according to the following scoring system: 0, no deficit; 1, forelimb flexion; 2, decreased resistance to lateral push; 3, unidirectional circling; 4, longitudinal spinning; and 5, no movement.

**Lung vascular permeability and water content assays**

For the assessment of lung vascular permeability, the extravasation of Evans Blue (EB) in the lung tissue was measured as described previously (Koma et al., 2014). A 2% solution of EB in 0.9% saline (500 mg/kg of body weight) was injected via tail vein and circulated for 60 minutes before euthanasia. The lungs were perfused free of blood with PBS containing 2 mM EDTA. For quantitative measurements, fresh lung tissue was weighed and immersed in 2 ml of N,N-dimethylformamide (Sigma), incubated for 18 hours at 55 °C. The supernatants were analyzed at 620 nm by spectrophotometry. The concentration of EB was then calculated according to established a standard curve. The lungs were placed in an incubator for 72 hours at 150 °C to obtain a constant weight. After the dry weight of tissue was recorded, the water content was calculated as= (wet weight-dry weight)/wet weight X 100%.

### **Bacteriological analysis**

Bacterial burden within the BALF and lung tissue was determined by brain–heart infusion (BHI) agar cultures as previously described (Stanley et al., 2016). At indicated time points after stroke, mice were euthanized and washed with 70% ethanol under sterile conditions. The trachea was surgically exposed and intubated with a syringe catheter. Bronchoalveolar lavage fluid (BALF) was obtained by instilling 0.7 ml pre-warmed PBS for three times. Then, the pooled lavage fluid was centrifuged (300 x g for 5 minutes). The cell pellet was counted and proceed for further flow cytometry analysis. The cell-free BALF was used for measuring protein concentration and bacterial load. After that, the lung tissues were removed, weighed and homogenized in sterile PBS (1:10, W/V). For determination of colony-forming units, 10 μl BALF or tissue homogenate was serially diluted, plated onto brain–heart infusion (BHI) agar plates supplemented with 5% sheep blood, and incubated for 18 hours at 37 °C, and then the bacterial colonies were counted by 2 researchers blinded to treatment groups.

**Lung histological and immunohistochemical Assessment**

At indicated time points after stroke, mice were euthanized and transcardially perfused with 0.01 M PBS pH 7.4, followed by 4% paraformaldehyde (PFA, Sigma). To collect lungs for histological examination, trachea was surgically exposed and cannulated (BD Angiocath). 0.5 ml of 4% PFA was injected through trachea to inflate lungs. Then, the inflated lungs were dissected and post-fixed overnight in excessive volume of 4% PFA, dehydrated, embedded in paraffin and sectioned using a microtome to yield 5 μm thick sections. Five sections from each animal were chosen, and stained with Haemotoxylin and Eosin (H&E) and mounted in Permount toluene solution (fisher scientific). The images were taken under a 20X magnification using Eclipse Ti-S Inverted Microscopes (Tokyo, Japan). The lung injury was evaluated by 2 blinded investigator and graded according to the following criteria as previously described (D'Alessio et al., 2009): 1, normal; 2, focal (<50% lung section) interstitial congestion and inflammatory cell infiltration; 3, diffuse (>50% lung section) interstitial congestion and inflammatory cell infiltration; 4, focal (<50% lung section) consolidation and inflammatory cell infiltration; 5, diffuse (>50% lung section) consolidation and inflammatory cell infiltration.

Immunohistochemistry was performed as described previously (Jin et al., 2011). Briefly, after deparaffinization and rehydration, sections were subjected to heat-induced epitope retrieval in 0.01mM citrate buffer (pH6.0). Nonspecific binding was blocked by treatment with blocking reagent for one hour at room temperature. The sections were then incubated with primary antibodies at 4 °C overnight. The primary antibodies were: goat anti-mouse MMP-9 (1:500; Cat: AF909, R&D Systems), rabbit anti-mouse PMN antibody (1:500; Cat: AIAG31140, Accurate Chemical). After primary antibody incubation, sections were washed in PBS for three times for 5 minutes. Then, sections were incubated with [Alexa Fluor® 488 Donkey Anti-Goat (IgG) and [Alexa Fluor® 555 Donkey Anti-Rabbit (IgG) secondary antibodies (1:500; Cat: ab150073 and ab150062, Abcam) for one hour at room tempreture.](https://www.abcam.com/donkey-rabbit-igg-hl-alexa-fluor-555-preadsorbed-ab150062.html)](https://www.abcam.com/donkey-goat-igg-hl-alexa-fluor-488-ab150129.html) After that, the sections were washed and mounted in Fluoroshield^TM^ with DAPI (Cat:F6057, Sigma). The images were then taken under a 20X magnification using Eclipse Ti-S Inverted Microscopes (Tokyo, Japan). All data were analyzed by two-blinded investigator.

**Flow cytometry**

At 24 hours after stroke, mouse blood samples were collected into heparinized tubes under deep anesthesia. After that, the mouse was transcardially perfused with 0.9% saline. Lung samples were collected and cut into small pieces (1-2 mm^3^), then digested in RPMI-1640 containing 2% FBS, type IV collagenase (1 mg/ml, Sigma-Aldrich) and DNase I (50 μg/ml, Roche Diagnostics) for 45 minutes at 37 °C under agitating (200 rpm) conditions, as previously described (Baral et al., 2018). The single-cell suspensions were passed through an 18-gauge needle ten times and filtered through a 70-μm cell strainer (Falcon). The cells were pelleted at 10 °C and 300 X g for 5 minutes. Red blood cells were lysed with RBC lysis buffer (eBioscience) and then resuspended in stain buffer (BD bioscience) and treated with Fc Block (rat anti-mouse CD16/CD32, 2.4G2). The single-cell suspensions were stained with antibody cocktails conjugated to the indicated fluorochromes or appropriate isotype controls (Suppl. table II). Cytofix/Cytoperm™ Plus Fixation/Permeabilization Kit (BD bioscience) was used for staining intracellular cytokines according to manufacturer’s protocol. Gates were set according to unstained samples, isotype controls and Fluorescence minus one (FMO) controls. Compensation was adjusted using BD CaliBRITE Beads (BD Bioscience). Flow cytometry was conducted on a BD Accuri™ C6 Plus (BD Bioscience). Data were obtained from three independent experiments and analyzed using FlowJo software (Treestar, version 10.0).

**Westen blot and Enzyme-Linked Immunosorbent Assays (ELISA)**

At 24 hours stroke, mouse blood samples were collected to heparinized tubes under deep anesthesia and processed to yield plasma. After that, the mouse was transcardially perfused with normal saline. Then, lung tissues were collected and homogenized with RIPA buffer containing protease inhibitors. Protein concentration in the supernatant and plasma was determined using a Bio-Rad protein assay. The concentrations of IL-17A and IFN-γ in lung tissue and plasma were measured using specific ELISA kits IL-17A and IFN-γ (purchased from Invitrogen and R&D system respectively) according to manufacturers’ protocol. Western blotting were performed as described previously (Jin et al., 2017). The primary antibodies was used as following: anti-CD147 (1:1000, Ab188190, Abcam), anti-MMP9 (1:1000, Ab38898, Abcam), and anti-β-actin (1:4000; A2066, Sigma) as loading control. Protein samples (20 μg protein each lane) were separated by SDS-PAGE and proteins transferred to polyvinylidene difluoride membrane. Membranes were blocked (5% nonfat milk in 0.05% PBS-Tween 20) and incubated with primary antibody (diluted in 3% bovine serum albumin in phosphate-buffered saline–Tween 20) overnight at 4°C. Membranes were then incubated with horseradish peroxidase-conjugated secondary antibody (in 5% milk in PBS-Tween 20). Immunopositive bands of horseradish peroxidase (HRP)-conjugated secondary antibodies were detected with an ECL blotting reagents (GE Healthcare) and developed with Chemiluminescent Western blotting detection Amersham Imager 600 system (GE Healthcare). Semi-quantitative assessment of immunoblots was performed by computerized densitometry.

**II. Supplemental References**

Agrawal SM, Silva C, Tourtellotte WW, Yong VW. (2011). EMMPRIN: a novel regulator of leukocyte transmigration into the CNS in multiple sclerosis and experimental autoimmune encephalomyelitis. *J Neurosci.* 31:669-677.

Agrawal SM, Silva C, Wang J, Tong JP, Yong VW. (2012). A novel anti-EMMPRIN function-blocking antibody reduces T cell proliferation and neurotoxicity: relevance to multiple sclerosis. *J Neuroinflammation.* 9: 64.

Baral P, Umans BD, Li L, Wallrapp A, Bist M, Kirschbaum T, et al. (2018). Nociceptor sensory neurons suppress neutrophil and γδ T cell responses in bacterial lung infections and lethal pneumonia. *Nat Med.* 24:417-426

Bederson JB, Pitts LH, Tsuji M, Nishimura MC, Davis RL, Bartkowski H. (1986). Rat middle cerebral artery occlusion: evaluation of the model and development of a neurologic examination. *Stroke.* 17:472–476.

[D'Alessio FR](https://www.ncbi.nlm.nih.gov/pubmed/?term=D%27Alessio%20FR%5BAuthor%5D&cauthor=true&cauthor_uid=19770521), [Tsushima K](https://www.ncbi.nlm.nih.gov/pubmed/?term=Tsushima%20K%5BAuthor%5D&cauthor=true&cauthor_uid=19770521), [Aggarwal NR](https://www.ncbi.nlm.nih.gov/pubmed/?term=Aggarwal%20NR%5BAuthor%5D&cauthor=true&cauthor_uid=19770521), [West EE](https://www.ncbi.nlm.nih.gov/pubmed/?term=West%20EE%5BAuthor%5D&cauthor=true&cauthor_uid=19770521), [Willett MH](https://www.ncbi.nlm.nih.gov/pubmed/?term=Willett%20MH%5BAuthor%5D&cauthor=true&cauthor_uid=19770521), [Britos MF](https://www.ncbi.nlm.nih.gov/pubmed/?term=Britos%20MF%5BAuthor%5D&cauthor=true&cauthor_uid=19770521), et al. (2009). CD4+CD25+Foxp3+ Tregs resolve experimental lung injury in mice and are present in humans with acute lung injury. [*J Clin Invest.*](https://www.ncbi.nlm.nih.gov/pubmed/?term=CD4%2BCD25%2BFoxp3%2B+Tregs+resolve+experimental+lung+injury+in+mice+and+are+present+in+humans+with+acute+lung+injury) 119:2898-2913.

Damsker JM, Okwumabua I, Pushkarsky T, Arora K, Bukrinsky MI, Constant SL. (2009). Targeting the chemotactic function of CD147 reduces collagen-induced arthritis. *Immunology.* 126: 55-62.

Gwinn WM, Damsker JM, Falahati R, Okwumabua I, Kelly-Welch A, Keegan AD, et al. (2006). Novel approach to inhibit asthma-mediated lung inflammation using anti-CD147 intervention. *J Immunol.* 177: 4870–4879.

Jin R, Song Z, Yu S, Piazza A, Nanda A, Penninger JM, et al. (2011). Phosphatidylinositol-3-kinase gamma plays a central role in blood-brain barrier dysfunction in acute experimental stroke. *Stroke.* 42: 2033-2044.

[Jin R](https://www.ncbi.nlm.nih.gov/pubmed/?term=Jin%20R%5BAuthor%5D&cauthor=true&cauthor_uid=29114092), [Xiao AY](https://www.ncbi.nlm.nih.gov/pubmed/?term=Xiao%20AY%5BAuthor%5D&cauthor=true&cauthor_uid=29114092), [Chen R](https://www.ncbi.nlm.nih.gov/pubmed/?term=Chen%20R%5BAuthor%5D&cauthor=true&cauthor_uid=29114092), [Granger DN](https://www.ncbi.nlm.nih.gov/pubmed/?term=Granger%20DN%5BAuthor%5D&cauthor=true&cauthor_uid=29114092), [Li G](https://www.ncbi.nlm.nih.gov/pubmed/?term=Li%20G%5BAuthor%5D&cauthor=true&cauthor_uid=29114092). (2017). Inhibition of CD147 (Cluster of differentiation 147) ameliorates acute ischemic stroke in mice by reducing thromboinflammation. [*Stroke.*](https://www.ncbi.nlm.nih.gov/pubmed/29114092) 48:3356-3365.

Jin R, Yu S, Song Z, Quillin JW, Deasis DP, Penninger JM, et al. (2010). Phosphoinositide 3-kinase-gamma expression is upregulated in brain microglia and contributes to ischemia-induced microglial activation in acute experimental stroke. *Biochem Biophys Res Commun.* 399:458-464.

[Koma T](https://www.ncbi.nlm.nih.gov/pubmed/?term=Koma%20T%5BAuthor%5D&cauthor=true&cauthor_uid=24719427), [Yoshimatsu K](https://www.ncbi.nlm.nih.gov/pubmed/?term=Yoshimatsu%20K%5BAuthor%5D&cauthor=true&cauthor_uid=24719427), [Nagata N](https://www.ncbi.nlm.nih.gov/pubmed/?term=Nagata%20N%5BAuthor%5D&cauthor=true&cauthor_uid=24719427), [Sato Y](https://www.ncbi.nlm.nih.gov/pubmed/?term=Sato%20Y%5BAuthor%5D&cauthor=true&cauthor_uid=24719427), [Shimizu K](https://www.ncbi.nlm.nih.gov/pubmed/?term=Shimizu%20K%5BAuthor%5D&cauthor=true&cauthor_uid=24719427), [Yasuda SP](https://www.ncbi.nlm.nih.gov/pubmed/?term=Yasuda%20SP%5BAuthor%5D&cauthor=true&cauthor_uid=24719427), et al. (2014). Neutrophil Depletion Suppresses Pulmonary Vascular Hyperpermeability and Occurrence of Pulmonary Edema Caused by Hantavirus Infection in C.B-17 SCID Mice. [*J Virol.*](https://www.ncbi.nlm.nih.gov/pubmed/?term=Neutrophil+Depletion+Suppresses+Pulmonary+Vascular+Hyperpermeability+and+Occurrence+of+Pulmonary+Edema+Caused+by+Hantavirus+Infection+in+C.B-17+SCID+Mice) 88(13):7178-7188.

Seizer P, Ochmann C, Schönberger T, Zach S, Rose M, Borst O, et al. (2011). Disrupting the EMMPRIN (CD147)-cyclophilin A interaction reduces infarct size and preserves systolic function after myocardial ischemia and reperfusion. *Arterioscler Thromb Vasc Biol.* 31:1377-1386.

[Stanley D](https://www.ncbi.nlm.nih.gov/pubmed/?term=Stanley%20D%5BAuthor%5D&cauthor=true&cauthor_uid=27694934), [Mason LJ](https://www.ncbi.nlm.nih.gov/pubmed/?term=Mason%20LJ%5BAuthor%5D&cauthor=true&cauthor_uid=27694934), [Mackin KE](https://www.ncbi.nlm.nih.gov/pubmed/?term=Mackin%20KE%5BAuthor%5D&cauthor=true&cauthor_uid=27694934), [Srikhanta YN](https://www.ncbi.nlm.nih.gov/pubmed/?term=Srikhanta%20YN%5BAuthor%5D&cauthor=true&cauthor_uid=27694934), [Lyras D](https://www.ncbi.nlm.nih.gov/pubmed/?term=Lyras%20D%5BAuthor%5D&cauthor=true&cauthor_uid=27694934), [Prakash MD](https://www.ncbi.nlm.nih.gov/pubmed/?term=Prakash%20MD%5BAuthor%5D&cauthor=true&cauthor_uid=27694934), et al. (2016). Translocation and dissemination of commensal bacteria in post-stroke infection. [*Nat Med.*](https://www.ncbi.nlm.nih.gov/pubmed/27694934) 22:1277-1284.

**Supplemental Tables**

**Suppl. Table I** Summary of experimental groups, treatments, and sample sizes

| **Group** | **Treatment** | **Animal No.** |
| --- | --- | --- |
| Sham | None | 55 |
| MCAO-24h and 72h | None | 16 |
| MCAO+isotype-24h | Isotype antibody (IV at 4h) | 42 |
| MCAO+αCD147-24h | αCD147 (IV at 4h) | 42 |
| MCAO+isotype-72h | Isotype antibody (IV at 4h, 24h and 48h) | 8 |
| MCAO+αCD147-72h | αCD147 (IV at 4h, 24h and 48h) | 9 |
| MCAO+isotype-72h | Isotype antibody (IV at 8h, 24h and 48h) | 6 |
| MCAO+αCD147-72h | αCD147 (IV at 8h, 24h and 48h) | 6 |

**Total 184**

**5**

**Suppl. Table II** Antibodies used for flow cytometric analysis

| **Antigen** | **Label** | **Clone** | **Isotype** | **Supply** |
| --- | --- | --- | --- | --- |
| CD16/CD32 | None | 2.4G2 | Rat IgG2b, κ | BD biosciences |
| CD45 | PerCP | 30-F11 | Rat IgG2b, κ | BD biosciences |
| CD147 | PE | OX-114 | Rat IgG1, κ | BioLegend |
| CD4 | APC | RM4-5 | Rat IgG2a, κ | BD biosciences |
| CD11b | APC | M1/70 | Rat IgG2b, κ | BD biosciences |
| CD41 | PE | MWReg30 | Rat IgG1, κ | BD biosciences |
| CD41 | FITC | MWReg30 | MWReg30 | BD biosciences |
| CD11c | PE | N418 | Armenian Hamster IgG | BioLegend |
| B220 | Alexa Fluor® 488 | RA3-6B2 | Rat IgG2a, κ | BioLegend |
| IAIE | FITC | M5/114.15.2 | Rat IgG2b, κ | BioLegend |
| Ly6G | APC | 1A8 | Rat IgG2a, κ | BD biosciences |
| Ly6G | FITC | 1A8 | Rat IgG2a, κ | BD biosciences |
| CD115 | APC | T38-320 | Rat IgG1, κ | BD biosciences |
| Ly6C | PE | AL-21 | Rat IgM, κ | BD biosciences |
| NK1.1 | FITC | PK136 | Mouse IgG2a, κ | BD biosciences |
| IL-17A | PE | TC11-18H10 | Rat IgG1, κ | BD biosciences |
| TCR-γδ | FITC | UC7-13D5 | Armenian Hamster IgG3, κ | eBioscience |
| IFN-γ | PE | XMG1.2 | Rat IgG1, κ | BD biosciences |


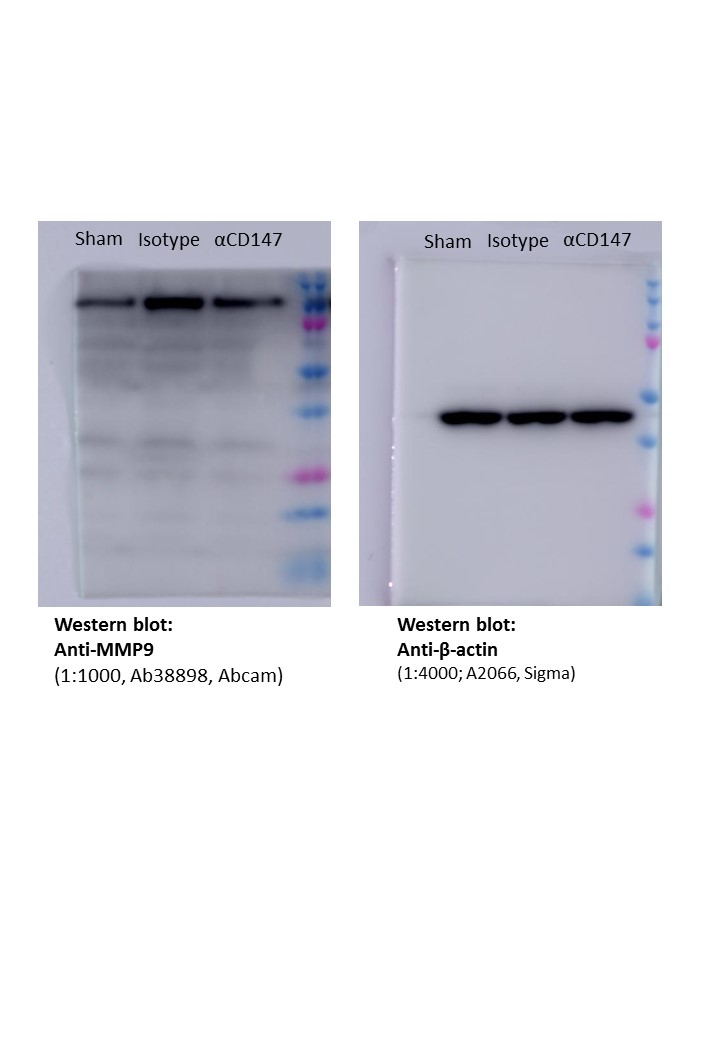

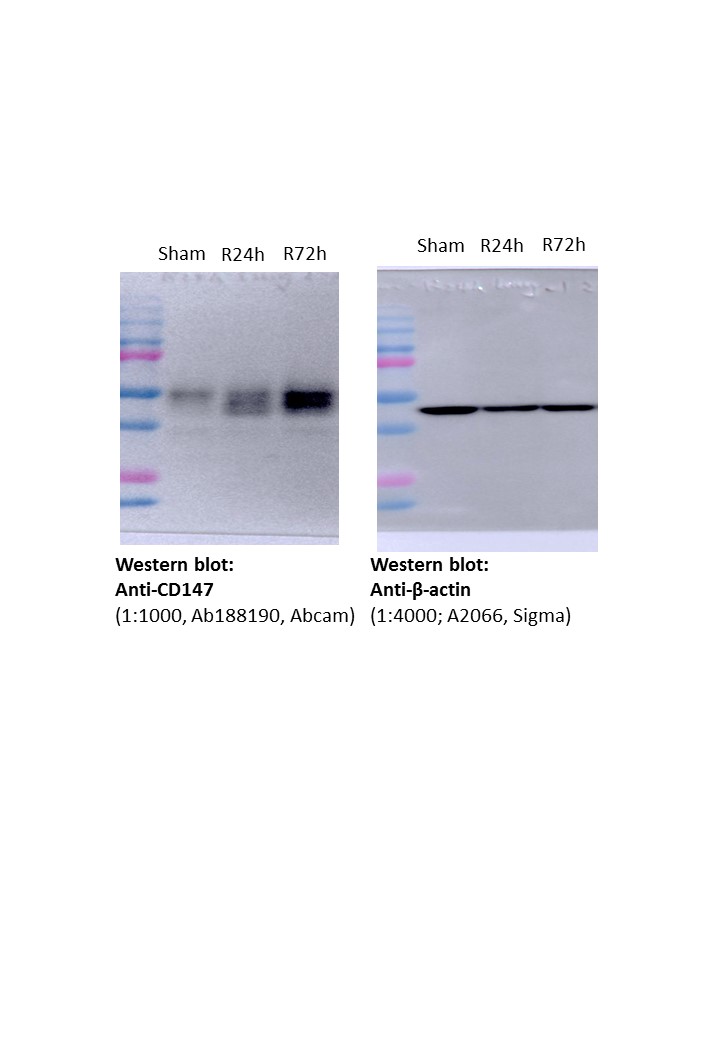


**Supple Figure 1:** The representative images of western blot. The mouse lung tissues were collected at 24 hours after stroke and homogenized in RIPA buffer with protease inhibitors. A total of 20µg protein was loaded in each lane.


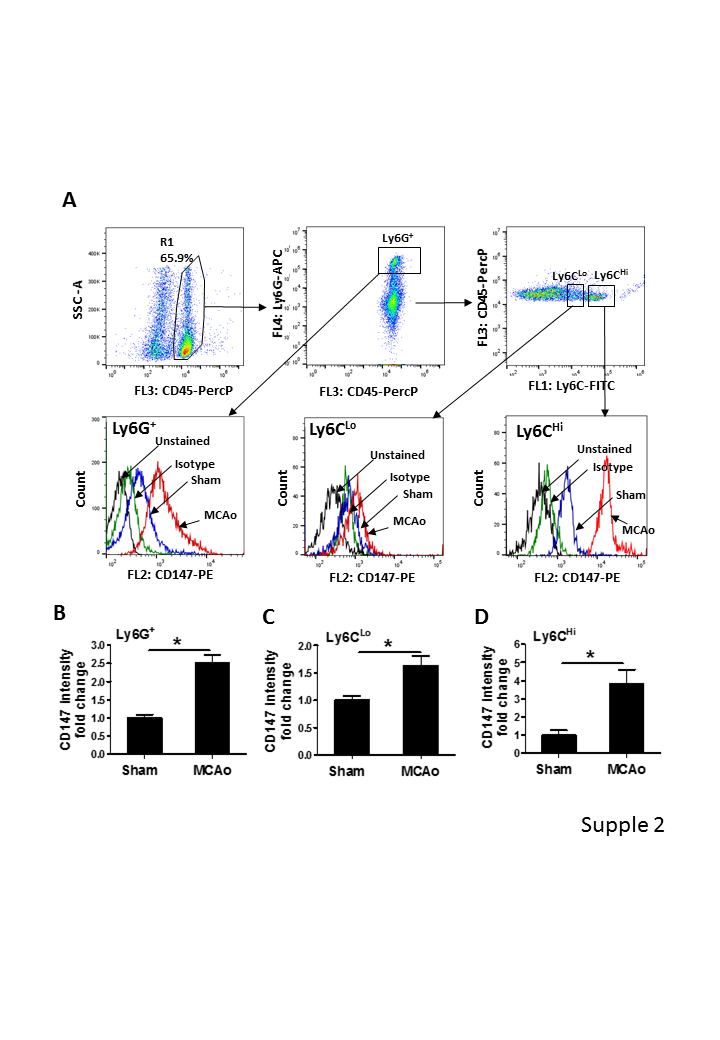


**Supple Figure 2: A,** Representative flow cytometry strategy for gating neutrophil, Ly6C^Lo^ and Ly6C^Hi^ monocyte/macrophage cell populations from isolated lung cells, histogram of flow cytometry showing CD147 expression on these cells in the indicated groups. **B, C and D**, Quantitative analysis of CD147 expression. n=4 per group.
